# The Brain Endothelial Cell Glycocalyx Plays a Crucial Role in the Development of Enlarged Perivascular Spaces in Obesity, Metabolic Syndrome, and Type 2 Diabetes Mellitus

**DOI:** 10.3390/life13101955

**Published:** 2023-09-24

**Authors:** Melvin R. Hayden

**Affiliations:** Department of Internal Medicine, Endocrinology Diabetes and Metabolism, Diabetes and Cardiovascular Disease Center, University of Missouri School of Medicine, One Hospital Drive, Columbia, MO 65211, USA; mrh29pete@gmail.com; Tel.: +1-573-346-3019

**Keywords:** blood–brain barrier, blood–cerebrospinal fluid barrier, blood–arachnoid barrier, endothelial glycocalyx, enlarged perivascular spaces, metabolic syndrome, neuroinflammation, obesity, perivascular spaces, T2DM

## Abstract

The brain endothelial cell (BEC) glycocalyx (ecGCx) is a BEC surface coating consisting of a complex interwoven polysaccharide (sweet husk) mesh-like network of membrane-bound proteoglycans, glycoproteins, and glycosaminoglycans (GAGs) covering the apical luminal layer of the brain endothelial cells. The ecGCx may be considered as the first barrier of a tripartite blood–brain barrier (BBB) consisting of (1) ecGCx; (2) BECs; and (3) an extravascular compartment of pericytes, the extracellular matrix, and perivascular astrocytes. Perturbations of this barrier allow for increased permeability in the postcapillary venule that will be permissive to both fluids, solutes, and proinflammatory peripherally derived leukocytes into the perivascular spaces (PVS) which result in enlargement as well as increased neuroinflammation. The ecGCx is known to have multiple functions, which include its physical and charge barrier, mechanical transduction, regulation of vascular permeability, modulation of inflammatory response, and anticoagulation functions. This review discusses each of the listed functions in detail and utilizes multiple transmission electron micrographs and illustrations to allow for a better understanding of the ecGCx structural and functional roles as it relates to enlarged perivascular spaces (EPVS). This is the fifth review of a quintet series that discuss the importance of EPVS from the perspective of the cells of brain barriers. Attenuation and/or loss of the ecGCx results in brain barrier disruption with increased permeability to proinflammatory leukocytes, fluids, and solutes, which accumulate in the postcapillary venule perivascular spaces. This accumulation results in obstruction and results in EPVS with impaired waste removal of the recently recognized glymphatic system. Importantly, EPVS are increasingly being regarded as a marker of cerebrovascular and neurodegenerative pathology.

## 1. Introduction and Background 

The brain endothelial cell (BEC) glycocalyx (ecGCx) is a BEC surface apical coating consisting of a complex polysaccharide (sweet husk) gel, a mesh-like network of membrane-bound proteoglycans, glycoproteins, and glycosaminoglycans (GAGs) covering the apical luminal surface of the BECs. Importantly, the ecGCx may be considered as a gatekeeper of BECs since it is important in maintaining physiological and homeostasis functions, which include the prevention of vascular permeability, inflammation, blood coagulation, and promoting the synthesis of the vasculoprotective nitric oxide (NO) via its mechano-sensing and transduction capabilities [1,2,3]. 

**Figure 1:**
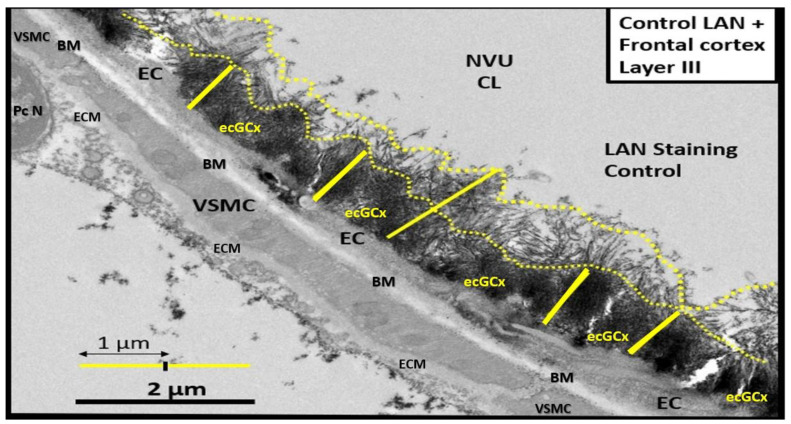
Transmission electron microscope (TEM) image of the brain endothelial cell glycocalyx (ecGCx) from a lanthanum nitrate (LAN)-stained postcapillary venule cortical layer, compared to the more dense staining of the ecGCx base core. The image displays how these apical projections can readily interact with proteins of the circulating plasma, proteins such as albumin, fibrinogen, and soluble PGNs that are capable of contributing to the repair of the ecGCx should it be damaged along with EC-synthesized reparative molecules. Also, note the unique brain endothelial cell (EC), which contains the tight and adherens junctions of the neurovascular unit (NVU). The blood–brain barrier (BBB) is covered by both an apical extracellular matrix (ECM) glycocalyx and basal ECM that forms the basement membrane-basal laminae (BM). Image obtained from previously published male CD-1, LPS treated, and LAN-perfused experiment [8]. Scale bar = 2 μm. *CL = capillary lumen; EC = brain endothelial cell; PcN = pericyte nucleus; VSMC = vascular smooth muscle cell*.

The ecGCx was first identified via transmission electron microscopy (TEM) by Luft utilizing ruthenium red staining to visualize the ecGCx in 1966 [4]. Recently, the ecGCx has been able to be identified using lanthanum (La(3+) nitrate (LAN) staining with perfusion fixation via transmission electron microscopy (TEM) specifically in the brain microvasculature neurovascular unit (NVU), blood–brain barrier (BBB) arterioles, precapillary arterioles, true capillaries, and postcapillary venules that has been both reliable and reproducible by this author and others (Figure 1) [5,6,7,8,9,10]. 

**Figure 2:**
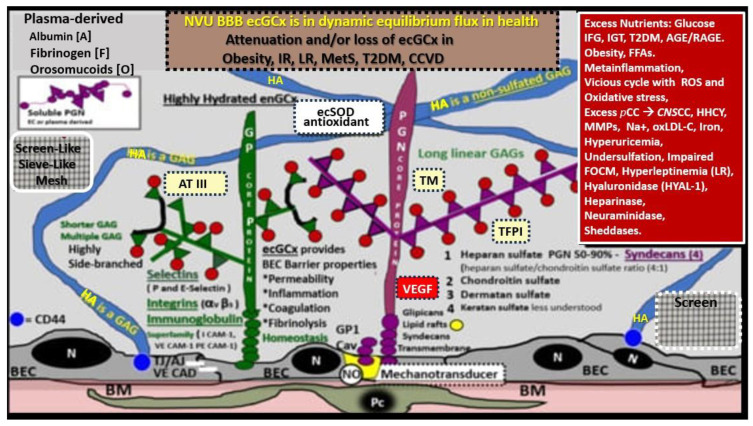
Structural components of the brain endothelial cell (BEC) glycocalyx (ecGCx) of the neurovascular unit (NVU) blood–brain barrier (BBB). This image depicts the individual components of the ecGCx, which is a unique apical BEC extracellular matrix. The ecGCx contains two classes of proteins that are primarily anchored, proteoglycan(s) (PGN) (of purple coloration) and glycoprotein(s) (GP) (of green coloration), and the glycosaminoglycans (GAGs) of hyaluronic acid (HA) hyaluronan (exceedingly long polymers up to micrometers in length and up to 40–60,000 kDa that are composed of uronic acid and hexosamine disaccharides (blue coloration). HA may be either free-floating (unattached), or attached to the CD44 cell BEC basilar plasma membrane, or form complexes of HA-HA. Non-sulfated HAs that are not anchored to the BECs may also reversibly interact at the lumen with plasma-derived albumin, fibrinogen, orosomucoids, and soluble PGNs [11,12]. The PGNs and GPs’ side chains consist of glycosaminoglycans (GAGs) covalently bound to core proteins that are highly sulfated (red dots) that contribute heavily to the net negative electrostatic charge of the ecGCx [11,13]. The two primary PGNs are syndecans and glypicans (purple). Glycoproteins (green) consist primarily of selectins (P and E), integrins (beta 3 and alpha v), and the immunoglobulin superfamily of ICAM-1, VE-CAM, and PE CAM. The BEC plasma membrane is where caveolar lipid rafts are located and contains CD44, which anchors hyaluronan (hyaluronic acid-HA), and glycosylphosphatidylinositol (GPI), that anchors glypican-1. Importantly, the GPI/glypican-1 interaction is thought to be responsible for mechanotransduction, which allows activation of endothelial nitric oxide synthase (eNOS) to synthesize vasculoprotective bioavailable nitric oxide (NO) via the calcium-calmodulin-dependent Caveolin-1 (Cav-1) protein. The soluble components of the ecGCx that are also important in the preservation of the important net negative electrostatic charge selectivity (orosomucoids and albumin) are derived from the bloodstream [11,13,14]. Note the upper right red box, which contains multiple factors that are known to be responsible for the perturbation of the ecGCx. Also, orosomucoids and the highly sulfated residues (red dots) are primarily responsible for the net negative change of the ecGCx [11,14]. Importantly, note the insertion of the mesh-like screen images, which give the ecGCx its sieving-like properties, that are placed to the left- and right-hand side of HA (blue). This illustration is not to scale. Images are modified with permission from CC 4.0 [6,7]. *AGE*/*RAGE = advanced glycation end-products and its receptor; aMt = aberrant mitochondria; ATIII = antithrombin III; CD44 = cluster of differentiation 44; CNS CC = central nervous system cytokines*/*chemokines; ecSOD = extracellular superoxide dismutase; FFAs = saturated free fatty acids; FOCM = folate-mediated one-carbon metabolism; HHCY = hyperhomocysteinemia; IFG = impaired fasting glucose; IGT = impaired glucose tolerance; IR = insulin resistance; LR = leptin resistance; ox-LDL-C = oxidized low-density lipoprotein-cholesterol; MMPs = matrix metalloproteinases; mtROS = mitochondrial reactive oxygen species; Na+ = sodium; N = nucleus; PCC = peripheral cytokine*/*chemokines; RAGE = receptor for AGE; RONSS = reactive oxygen, nitrogen, sulfur species; ROS = reactive oxygen species; RSI = reactive species interactome; T2DM = type 2 diabetes mellitus; TFPI = tissue factor pathway inhibitor; TJ*/*AJ = tight and adherens junctions; TM = thrombomodulin;; VE CAD = vascular endothelial cadherin*.

Even at the ultra nano-structure level the net negatively charged ecGCx stains are so intensively electron-dense, due to the ecGCx attracting the highly positively-charged lanthanum La(3+) nitrate (LAN) of the electron-dense staining lanthanum nitrite, that the proteins cannot be seen. Therefore, the following illustration is utilized in order to better understand and illustrate the proteoglycan, glycoproteins, and GAGs including hyaluronan (HA). Also, the GAGs covalently bonded to proteoglycans and glycoproteins’ side chains with their large quantity of negatively charged sulfate residues are included (Figure 2) [6,7,11,12,13,14].

**Figure 3:**
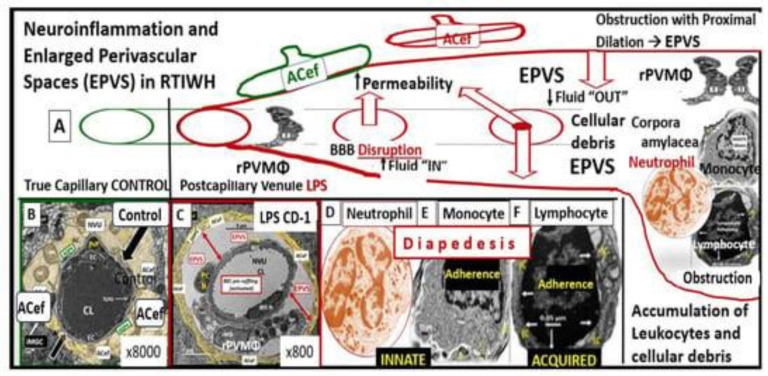
Perturbation of the endothelial glycocalyx results in brain injury to the brain endothelial cells (BECs) with activation of the response to injury wound healing mechanism that results in remodeling, neuroinflammation, and enlarged perivascular spaces (EPVSs). Panel (**A**) depicts the natural progression of the perivascular space (PVS) as it undergoes transformation or remodeling to an EPVS (outlined in red) in a linear fashion from the left- to right-hand side of this image (outlined in red). Panel (**B**) depicts transmission electron microscopic (TEM) images of a true capillary without a pia mater lining or PVS in a healthy control 20-week-old female model. Panel (**C**) depicts an EPVS that contains a resident, reactive, and migratory perivascular macrophage (rPVMΦ) obtained from a 7-week-old female lipopolysaccharide (LPS)-treated model. Panels (**D**–**F**) depict labeled leukocytes that undergo diapedesis across the disrupted BBB neurovascular unit (NVU) brain endothelial cells (BECs), which enter the newly remodeled EPVSs that interact with the rPVMΦs. These leukocytes that have entered the PVSs are capable of generating excessive phagocytic cellular debris due to reactive oxygen species and proteases generated by reactive leukocytes. Further, this increase in reactive leukocytes and rPVMΦs along with their excessive cellular debris result in obstruction of the PVS, which result in the dilation of the PVS proximal to the obstruction, creating the EPVS as observed on magnetic resonance imaging (MRI), such as those that occur in the basal ganglia and centrum semiovale structures in the white matter, and post-capillary venules that are important in the efflux of waste materials to the cerebrospinal fluid in cerebral small vessel disease and neurodegenerative diseases. Panels (**A**–**F**) depict the two-step process to neuroinflammation, while panel (**A**) only depicts step one comprised of leukocyte transmigration from the disrupted blood–brain barrier (BBB) into the PVS of a two-step process. Step two is known to allow for the incorporation of the generated reactive oxygen species and matrix metalloproteinases (MMP2, MMP9) from reactive leukocytes, which are capable of digesting the glia limitans basement membrane barrier of the outermost boundary of the EPVS created by the lining perivascular astrocytes. Subsequently this will allow for the transmigration of leukocytes from the EPVS to enter the interstitial spaces of the parenchymal neurons to complete the neuroinflammation remodeling mechanism that is present in obesity, metabolic syndrome, and type 2 diabetes mellitus. This image has been modified with permission from CC 4.0 [19]. *ACef = astrocyte endfeet; CL = capillary lumen; EPVS = enlarged perivascular space; rPVMΦ = resident perivascular macrophage*.

Previously, the brain endothelial cells have been examined as central players in the development of EPVS and as a result, this also places the ecGCx at a critical point in the development of EPVS [15]. The ecGCx is known to have multiple actions/functions including its physical and charge barrier, mechanical transduction, regulation of vascular permeability, modulation of inflammatory response, and anticoagulation functions [16,17]. The ecGCx plays a critical role and is also a critical component of the extended neurovascular unit that not only influences the structure and function of the BBB but also performs multiple physiological functions including maintaining normal neuronal homeostasis [18]. The first line of defense or barrier property against BEC injury is the ecGCx which performs a vital and critical role in maintaining brain homeostasis against BEC injury. When there is an attenuation, loss, or a discontinuous ecGCx of the NVU, the BBB will undergo disruption and allow increased NVU permeability. Interruption of the first barrier of the tripartite barrier (consisting of (1) ecGCx; (2) BECs; (3) extravascular compartment will allow for increased permeability in the postcapillary venule that will be permissive to both fluids and proinflammatory peripherally derived leukocytes in the perivascular space (PVS), resulting in enlargement as well as increased neuroinflammation (Figure 3) [6,7,18,19]. 

**Figure 4:**
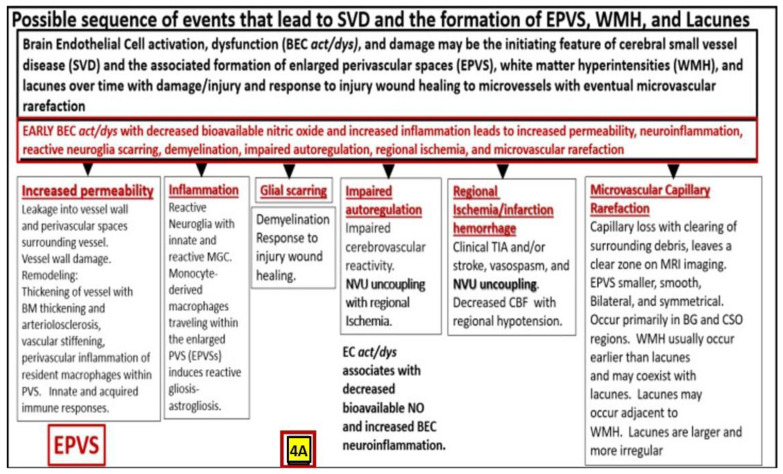

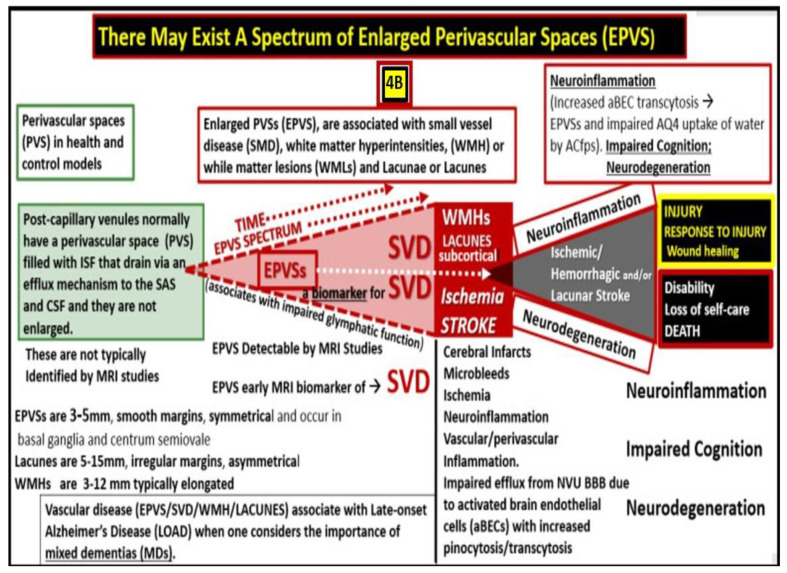
A likely sequence of events and an ongoing spectrum of development for enlarged perivascular spaces (EPVS). Brain endothelial cell activation, dysfunction, and damage may be the initiating factor in this sequence of events in figure four, while attenuation and/or loss of the endothelial glycocalyx may be concurrent or even precede it. Also, there exists a sequence of remodeling changes and events in figure four crucial in the development of EPVS, white matter hyperintensities, lacunes and small vessel disease. Images provided with permission from CC 4.0 [19,20,28]. *ACfp = astrocyte endfeet; BBB = blood–brain barrier; BEC = brain endothelial cell; BEC act*/*dys = brain endothelial cell activation and dysfunction; BG = basal ganglia; BM = basement membrane; CBF = cerebral blood flow; CSF = cerebrospinal fluid; CSO = centrum semiovale; ISF = interstitial fluid; LOAD = late-onset Alzheimer’s disease; MGC = microglia cell; mm = micrometer; MRI = magnetic resonance imaging; NO = nitric oxide; NVU = neurovascular unit; PVS = perivascular spaces; spaces; SAS = subarachnoid space; TIA = transient ischemic attack. WMH = white matter hyperintensities*.

EPVS are thought to have numerous causes from multiple mechanisms including: (1) Increased fluid and neurotoxic proteins that enter the PVS due to BBB dysfunction/disruption as a result of increased permeability. (2) Increased fluid inflow into the PVS due to increased BBB permeability as a result of BBB disruption and concurrent astrocyte endfeet (ACef) dysfunction, detachment, separation, and aquaporin-4 dysfunction with decreased water uptake allowing for the accumulation of water in the PVS. (3) The stalling or obstruction of impaired glymphatic efflux of the PVS conduit due to inflammation as a result of the accumulation of excess leukocytes with phagocytosis and the accumulation of excessive phagocytic debris and oxidative stress, in addition to the activation of increased matrix metalloproteinases (MMPs) that result in stagnation, stalling, and/or varying degrees of PVS conduit glymphatic system obstruction of the waste removal mechanisms. (4) Microvascular arteriole or venule vascular stiffening and/or aberrant spiraling of arterioles that are associated with decreased vascular pulsatility, which result in decreased fluid flow within the PVS, which contributes to PVS enlargement (mechanisms [1,2,3,4] that were discussed in Figure 3 and its legend previously). Or (5) neuronal atrophy or apoptosis surrounding neurons or their axons [19,20,21,22,23,24,25,26,27,28]. Notably, EPVS do not develop all at once, but are thought to be associated with a sequence of events and exist as in an evolutionary spectrum, such that they develop gradually to result in SVD, neuroinflammation, impaired cognition, and neurodegeneration (Figure 4A,B) [19,20,28].

EPVS are recognized as important aberrant structural remodeling changes in various neuropathologies and are currently known as biomarkers for cerebral small vessel disease (SVD) in addition to vascular dementia (VaD). Additionally, SVD and VAD are known to be associated with lacunar stroke and white matter hyperintensities (WMH) [20,22,23,24,25,26,29]. Notably, EPVS are associated with hypertension, advancing age, intracerebral hemorrhages, microbleeds, lacunes, cerebrocardiovascular diseases, transient ischemic episodes and stroke, SVD, cerebral autosomal dominant arteriopathy with subcortical infarcts and leukoencephalopathy (CADISIL), cerebral amyloid angiopathy (CAA), metabolic syndrome (MetS), obesity, type 2 diabetes mellitus (T2DM), WMH, sporadic Alzheimer’s and Parkinson’s disease, and non-age-related multiple sclerosis [20,21,22,23,24,26,27,29,30,31,32,33,34,35]. Further, our global population is already one of the oldest in history and it is only expected to continue to age [5]. Since it is known that EPVS are associated with aging it is felt that EPVS will continue to be more prevalent and are expected to grow in the coming years [20,36]. Additionally, EPVS are related to extracranial atherosclerosis, cerebromacrovascular and cerebromicrovascular disease [36] in addition to age-related neurodegenerative diseases such as sporadic Alzheimer’s and Parkinson’s disease.

The ecGCx is known to maintain normal neuronal homeostasis. It acts specifically as a physical and charge barrier, a mechanical transducer, a regulator of vascular permeability, a modulator of inflammatory response, and in anticoagulation [11,16]. An intact glycocalyx is necessary for the maintenance, stability, and integrity of the internal environment of the brain. Thus, damage to the ecGCx is capable of resulting in the dysfunction of these barriers as a result of this disruption [16]. 

The general methods for this narrative review consisted of an in-depth literature search that mainly focused on the role of the ecGCx, PVS/EPVS, neurovascular, neuroinflammatory and neurodegenerative mechanisms, which was performed utilizing electronic databases. Experimental methods utilized throughout this review to study the brain ecGCx and provide representative figure images include the following: LAN-perfused fixation and TEM studies of the obese, insulin- and leptin-resistant, and diabetic female BTBR *ob*/*ob* genetic model at 20 weeks of age [6] and the lean, male, non-diabetic CD-1 at 7 to 8 weeks of age treated with LPS and additionally perfusion fixed with LAN to provide a neuroinflammatory model [8]. The details regarding animals, material and methods, tissue collection and preparation for TEM, analysis, and statistics can be viewed in the following references: [6,8]. Additionally, each of these models were called out in their respective figure image legends and text. 

## 2. The Endothelial Glycocalyx (ecGCx) Structure 

**Figure 5:**
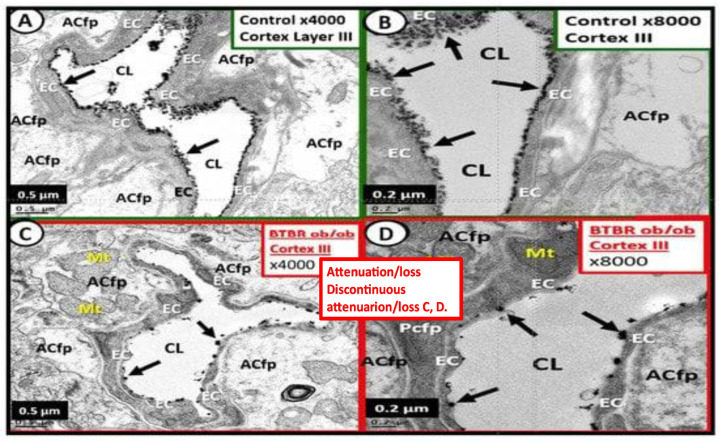
These electron micrographs depict the attenuation, loss, or discontinuous brain endothelial cell glycocalyx (ecGCx) in lanthanum nitrate-stained, obese, insulin-resistant, diabetic BTBR *ob*/*ob* at 20 weeks of age in female mouse models. Panels (**A**,**B**) are from the normal control model (heterozygous BTBR *ob*/*ob*) and demonstrated the normal lanthanum staining of the ecGCx and note the continuous electron-dense nature of this surface coating layer with lanthanum. Panels (**C**,**D**) depict the small black discontinuous dots (clumping) and attenuation in contrast to the continuous ecGCx in control models. These remodeling changes would contribute to increased permeability at the NVU BBB to allow the passage of fluid and inflammatory leukocytes into the perivascular spaces to result in enlargement. Thus, these findings rendered the BBB disrupted due to perturbations of its ecGCx. Reproduction of this modified image is permitted by CC 4.0 [6].

The ecGCx is a nanosized structure making it difficult to study with light microscopy. Only recently has lanthanum nitrate become more widely available to allow for TEM studies to supplement Lufts’ earlier work with ruthenium red to stain the ecGCx utilizing TEM in 1966 [4]. Most recently, the author and Ando et al. [6,8,10] have shown that LAN staining and studies are both reliable and reproducible due to their strong electrostatic attraction as in Figure 1. Notably, the obese, insulin-resistant, and diabetic DTBR *ob*/*ob* female mouse model at 20 weeks of age revealed that the NVU BECs had an attenuation, loss, and/or became discontinuous with LAN perfusion fixation and staining (Figure 5) [6].

**Figure 6:**
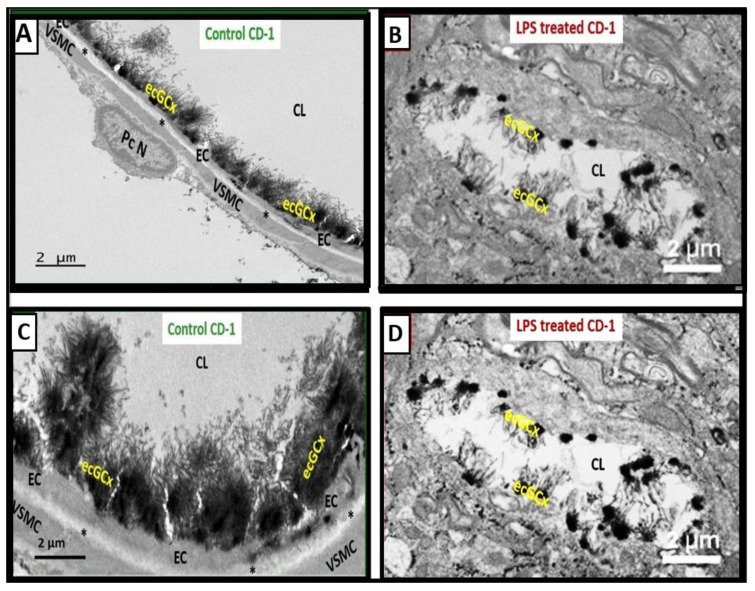
Attenuation, loss, or discontinuous brain endothelial cell glycocalyx (ecGCx) in lipopolysaccharide (LPS)-treated non-diabetic male CD-1 model at seven weeks of age with lanthanum nitrite (LAN) staining. Panels (**A**,**C**) demonstrate the normal electron-dense LAN staining of the ecGCx in control non-LPS treated, LAN-perfused mice. Panels (**B**,**D**) treated with LPS depict the attenuation, loss, or discontinuous ecGCx, which would allow for neurovascular unit (NVU) blood–brain barrier (BBB) disruption with increased permeability to fluids, cytokines and chemokines, cells, and in particular inflammatory leukocytes. These could then pass into the postcapillary venular perivascular spaces (PVS) in obese, insulin-resistant, diabetic BTBR ob/ob mice at 20 weeks of age. Notably the increased fluid and proinflammatory cellular entry could enlarge the PVS to the point of enlargement and sustain this enlargement. Note that LAN staining interferes with normal staining of the surrounding membranes in some images. These representative images have not been previously published but were from the experimental study [8]. Scale bars = 2 μm. *Asterisk = basement membrane of brain endothelial cell; CL = capillary lumen; EC = brain endothelial cell; PcN = pericyte nucleus; VSMC = vascular smooth muscle cell*.

Additionally, the following representative image is from a male LPS-treated male CD-1 mouse model at seven weeks of age (Figure 6) [8]. 

LAN-perfused images in Figure 6 only had an n = 2; therefore, the data collected were too small to perform any type of statistical studies in the original experiment [8]. However, these images were only meant to be representative of the perturbation of the ecGCx in LPS-treated neuroinflammatory models. Sampie et al. studied obese, insulin- and leptin-resistant, and diabetic *db*/*db* male mouse models at 10 weeks of age treated with LPS and stained via LAN perfusion fixation [37]. This group found that the ecGCx was already thinned in the diabetic *db*/*db* models compared to controls and that LPS treatment resulted in further thinning and even shedding in the pulmonary endothelium. Additionally, they also found that LPS treatment delayed the migration of inflammatory cells as well as expanding them and hypothesized that this extended inflammation may be involved in even further ecGCx damage [37]. Also, Erickson et al. demonstrated in a recent study that 7–8-week-old male CD-1 LPS-treated mice had pronounced ultrastructural remodeling changes in BECs that included plasma membrane ruffling, increased pinocytosis, transcytosis, increased numbers of extracellular microvesicles, small exosome formation, and aberrant BEC mitochondria, while TJ/AJ appeared to be intact. Also, Pc were contracted with rounded nuclei and loss of their elongated cytoplasmic processes (retraction). Interrogating microglia cells were attracted to the NVU, and astrocyte detachment and separation were associated with the formation of perivascular spaces and pericapillary edema—EPVS [8]. 

## 3. ecGCx Acts as a Physical Barrier

The ecGCx is a molecular sieve with extensive interlacing of its constituent molecules (Figure 2), which limits the exposure of multiple proteins including adhesion molecules such as intercellular adhesion molecule-1/vascular cell adhesion molecule-1 (ICAM-1/VCAM-1), von Willebrand factor, antithrombin, tissue factor pathway inhibitors, NO synthase, and extracellular superoxide dismutase [11]. In addition, the ecGCx excludes leukocytes, erythrocytes, plasma cells and platelets such that they cannot easily penetrate the ecGCx [11,16,17]. Subsequently, a complete and stable ecGCx inhibits the interaction not only between molecules but also cells.

## 4. The ecGCx Acts as an Electrostatic Charge Barrier 

The high sulfation of the GAGs within the ecGCx, orosomucoids, and albumin interact to create a net negative electrostatic charge and allow for the electrostatic repulsion of negatively charged molecules, while promoting the facilitation of positively charged molecules due to electrostatic charge repulsion and absorption (Figure 1 and Figure 2) [11,38,39,40]. 

## 5. The ecGCx as a Mechanosensor 

Blood pressure and shear stress affect the ecGCx and are capable of inducing changes within the BECs to affect its cytoskeleton and result in increased eNOS and bioavailable NO synthesis to cause vasodilation and increase flow corresponding to increased pressure and shear stress [41]. This is thought to be largely due the interaction with glycosylphosphatidylinositol (GPI), which anchors glypican-1 to allow for the activation of endothelial nitric oxide synthase (eNOS) to synthesize bioavailable NO via the calcium-calmodulin-dependent caveolin-1(CAV-1) protein [6,7,16,39,41]. Notably, vascular permeability is modified by the molecular size, structure, and especially the electrostatic charge of the ecGCx [16,39,41,42].

## 6. The ecGCx as a Regulator of Permeability 

Paracellular tight and adherens junctions (TJ/AJs) remain the main proteins involved in the control of NVU BEC permeability; however, in the past decade the ecGCx has also been recognized as a key regulator of permeability [18]. Previous observations have suggested that the main osmotic pressure gradient opposite to fluid filtration begins with ecGCx, but the effect of the interstitial protein concentration was small [43]. Importantly, the ecGCx is now considered to be the first barrier of a three-part BBB with the BEC as the second barrier and the abluminal BEC BM and ACef (considered the extravascular compartment) as its third barrier [18]. Thus, the ecGCx is a major permeability barrier of BECs [42,43,44].

## 7. The ecGCx as a Regulator of Inflammation 

The ecGCx heparan sulfate proteoglycans (HSPGs) are known to serve as a ligand for certain selectins and chemokines and play an important role in the turnover of leukocytes at the postcapillary venules [45]. Chronically excessive peripherally derived cytokines and chemokines and their dysregulation are a central feature in the development of neuroinflammation, neurodegeneration, and impaired cognition [6]. Since excessive cytokines/chemokines eventually result in ecGCx attenuation and/or shedding this protective effect will be lost such that inflammation begets inflammation and a vicious cycle will ensue and herein lies the problem in obesity, MetS, and T2DM. Additionally, the ecGCx is able to (1) shield BECs from integrins and other molecules that stimulate the binding of proinflammatory leukocytes to the endothelial plasma membrane surface coat as well as interfering with antigen presentation and T cell activation; (2) create a regional chemokine gradient that favors leukocyte activation; (3) regulate the activity of chemokines, cytokines, and growth factors by protecting them from enzymatic degradation [16]. Notably, a healthy ecGCx provides steric hindrance of receptor/ligand binding during leukocyte adhesion to the endothelium and is known to regulate inflammation due to various causes via different mechanisms [46], which include its shielding effects from integrins and co-stimulating molecules that are important in inhibiting the binding of proinflammatory leukocytes to endothelial surfaces. For example, glycosaminoglycans interact selectively with chemokines (such as RANTES, CxCL1, MCP-1, IL-8, and MIP1alpha) and modulate their receptor binding and cellular responses [47], their mechanisms of inhibiting antigen presentation and T cell activation, their protective mechanisms of cytokines and growth factors from enzymatic degradation, and their mechanisms of binding regulatory proteins and aiding in complementing inhibition [48]. 

**Figure 7:**
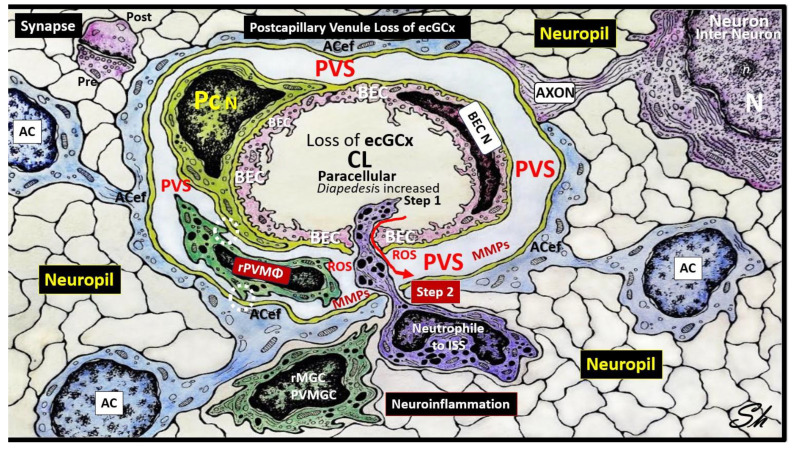
Loss or attenuation of the endothelial glycocalyx (ecGCx) in the postcapillary venule results in blood–brain barrier disruption and increased permeability. Note the red arrow denoting that there will be increased fluid entering the perivascular space (PVS) as well as the transmigration of proinflammatory leukocytes into the PVS via step one, and also note the breech of the PVS as depicted by the purple-colored neutrophile via the outermost basal laminae of the astrocyte endfeet (ACef) into the interstitial space of the neuronal parachyma via step one resulting in neuroinflammation. Importantly, note the red arrow denoting the loss of fluid and solutes from the capillary lumen to the perivascular space with enlargement. Additionally, tight and adherns junctions will become aberrant to allow for a paracellular breech. Importantly, note the complete loss of the brain endothelial cell (BEC) glycocalyx in this depicted neurovascular unit capillary. *AC = astrocyte; ACef = astrocyte endfeet; BEC = brain endothelial cell; ISS = interstitial space; MMPs = matrix metalloproteinases; N = nucleus; Pc N = pericyte nucleus; rPVMΦ = resident, reactive perivascular macrophage; ROS = reactive oxygen species*.

In healthy homeostatic conditions, true capillaries without pia mater and PVS are important for the uptake of water, oxygen, nutrients, and metabolic efflux while the post-capillary venule and its PVS are responsible for the transmigration of immune cells (innate and adaptive leukocytes) into the CNS parenchyma as previously described by Owens et al. [49]. Importantly, Owens et al. have shared the concept of the two steps to neuroinflammation wherein step one consists of the proinflammatory leukocytes breeching the BBB (due to BBB disruption via perturbation of the ecGCx and dysfunctional or damaged TJ/AJ) and entering into the PVS and step two consists of PVS proinflammatory leukocytes breaching the outer basement lamina of the perivascular space (via perivascular space proinflammatory-derived oxidative stress and MMP damage to the astrocyte basal lamina) to allow proinflammatory leukocytes to enter the ISS of the neuronal parachyma upon transmigration from the PVS (Figure 7) [49]. 

Notably, PVS serve as a conduit for the efflux of waste to the SAS and CSF to the dural venous sinus and the systemic circulation, which is now called the glymphatic system, initially described by Iliff and others [50]. 

## 8. The ecGCx as an Anticoagulant 

The ecGCx physical barrier prevents the blood cells and endothelium from coming into direct contact and thus avoids coagulation [11]. The ecGCx is a significant physiological binding site for anticoagulant factors, the tissue factor pathway inhibitor (TFPI), and antithrombin III (AT III) [11,51]. Furthermore, antithrombin III combines with HSPGNs to enhance its anticoagulant effect and thrombomodulin can bind to chondroitin sulfate that converts thrombin into an activator of the protein C pathway to strengthen the anticoagulant pathway [11]. The tissue pathway inhibits FVIIa (Hageman factor) and FXa (xabans) and interacts with HSPGs and the ecGCx [11,52]. Additionally, TFPI is a Kunitz-type protease inhibitor that inhibits the initial reactions of blood coagulation which resides in the ecGCx and forms a major pool of TFPI [53]. Additionally, thrombomodulin (TM) resides within the ecGCx and provides natural anticoagulant properties to the endothelium by inhibiting thrombin [54,55]. These previous actions of the ecGCx summarily contribute to the natural inhibition the coagulation process as in (Figure 2). 

## 9. The Importance of the Brain Barriers/Interfaces 

**Figure 8:**
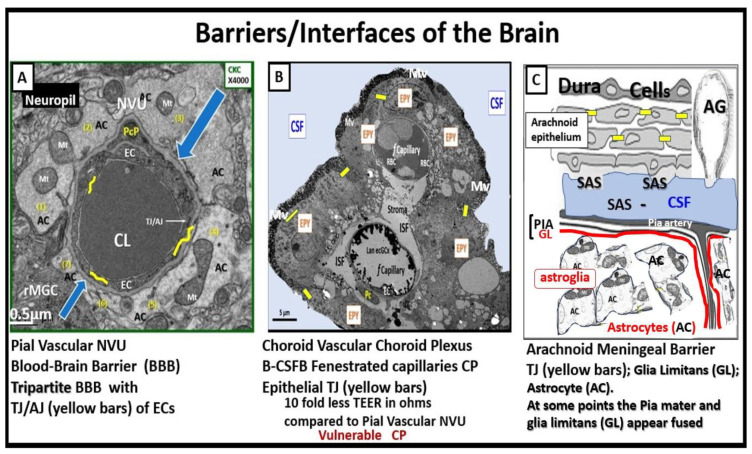
Three barriers/interfaces of the brain. Panel (**A**) demonstrates a transmission electron microscopic (TEM) image of a control healthy pia artery-derived blood–brain barrier (BBB) from the female C57B6 J model at 20 weeks of age. Note that tight and adherens junctions (TJ/AJ) are pseudo-colored with yellow lines, and blue open arrows point to the basement membrane. Panel (**B**) depicts a choroid artery-derived TEM image of a lanthanum nitrite (LAN)-perfused fixed staining of an LPS-treated male CD-1 model of the blood-cerebrospinal fluid barrier (B-CSFB) at 7 weeks of age and note the positive intense electron negative staining of the lower fenestrated capillary with LAN-stained endothelial glycocalyx (ecGCx) without microthrombi. Also, it is necessary to point out that in the upper capillary without LAN-positive staining the ecGCx contains microthrombi. Additionally, note that only vascular stromal cells and not astrocytes surround the fenestrated capillaries of B-CSFB. Panel (**C**) presents a hand-drawn illustration of a normal healthy arachnoid barrier of the meninges (not to scale). Panels (**A**,**B**) are provided with permission from CC 4.0 [6]. *AC = astrocyte(s); AG = arachnoid granulations; CKC = control model; CL = capillary lumen; CP = choroid plexus; CSF = cerebrospinal fluid; EC(s) = brain endothelial cell(s); EPY = epithelial ependyma cells; fCapillary = fenestrated capillary; ISF = interstitial fluid; Mv = microvilli of the ependymal cells; Mt = mitochondria; NVU = neurovascular unit; PcP = pericyte process; rMGC = reactive microglial cell; SAS = subarachnoid space; TEER = trans-epithelial electrical resistance; TJ = tight junction(s); TJ*/*AJ = tight and adherens junction*.

While this review has focused heavily on the BBB with tight and adherens junctions (TJ/AJ) with their claudins, occludins, and junctional adherens molecules (JAMs) and adherens junctions (VE-cadherins) there are actually three main barriers/interfaces in the CNS [56,57,58]. In addition to the BBB there are two other important barriers that should be discussed, namely the blood–cerebrospinal fluid barrier (B-CSFB) and the meningealarachnoid barrier, which is also a B-CSFB barrier (Figure 8) [57,58,59,60]. 

Notably, the B-CSFB fenestrated capillaries are stained heavily with LAN in control models with perfusion-fixed staining in contrast to the LPS-treated models (Figure 8B). Additionally, the B-CSFB is also present at the median eminence of the third ventricle lined by the epithelial tanycytes and the circumventricular organs of the hypothalamic nuclei [61]. Also, the arachnoid barrier, which is a component of the B-CSFB, is located in the meninges and is composed of arachnoid epithelial cells with tight junctions (Claudin 11) and adherens junctions (E-Cadherins) (Figure 8C) [62]. 

The choroid plexus (CP) and its B-CSFB are critical for maintaining brain homeostasis. Its aberrant function and increased permeability have been recently associated with different diseases of the CNS including neurodegenerative diseases such as Alzheimer’s disease and autoimmune diseases such as multiple sclerosis. Also, the CP/B-CSFB has been shown to be important in restoring homeostasis after stroke and brain injury [63,64].

## 10. Possible Mechanisms of Action Regarding How Attenuation and/or Loss of ecGCx Contributes to the Development of Enlarged Perivascular Spaces

When the ecGCx becomes attenuated and/or lost the NVU BBB is known to become disrupted and develops increased permeability with the leakage of water, solutes, plasma proteins, and proinflammatory leukocytes, peripheral cytokines/chemokines, ROS, and MMPs (Figure 7) [65]. The loss of the ecGCx also leads to a destabilization of the adjacent BEC actin cortical web, which in turn is known disrupt junctional (tight and adherens junctional) protein alignment and prevent intercellular communication between adjacent ECs. Upon this destabilization of the tight and adherens junctions this paracellular junction will develop increased permeability to proinflammatory leukocytes, plasma, and plasma proteins and other neurotoxicities [66].

PVS are critical conduits for interstitial fluid and metabolic waste into the CSF [67], and EPVS efflux could be negatively affected by compromised BBB integrity and associated BEC inflammation [68]. As noted in previous figures (Figure 7 and Figure 8A), the NVU BBB is a selective barrier composed of a true capillary, postcapillary venule BEC, TJ/AJ, pericytes, and astrocytes [69]. The excess accumulation of the peripheral proinflammatory leukocytes, including increased peripherally derived monocyte-derived macrophages, that become perivascular macrophages and the antigen presentation by pericytes, BECs, and rPVMΦ proteases (MMPs), cytokines/chemokines, and oxidative stress will result in excessive phagocytic debris within the PVS. Thus, this accumulation of excessive debris and stagnation of flow within the EPVS will result in impaired efflux within the EPVS or glymphatic system allowing neurotoxins to accumulate and not be cleared [20,23]. Additionally, this accumulated neurotoxic debris will increase inflammation and further aggravate the dilation of the EPVS. In addition to neuroinflammation affecting the integrity of the paracellular TJ/AJs between BECs, increased BBB permeability will also affect endothelial function that also relates to SVD. As a result of these remodeling changes, EPVS are not only considered a biomarker for SVD but also a possible marker for BBB disruption or dysfunction, and impaired glymphatic efflux [20,23].

## 11. Conclusions 

**Figure 9:**
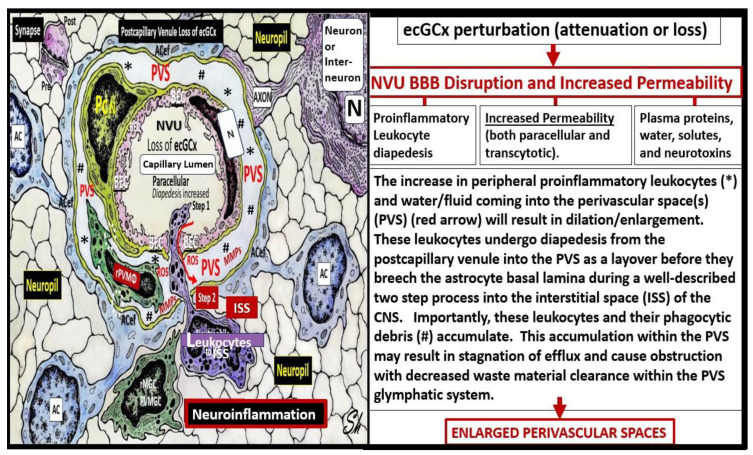
Possible mechanism of action indicating how the loss of the brain endothelial glycocalyx (ecGCx) results in enlarged perivascular spaces (EPVS). This illustration demonstrates that when there is an attenuation and/or loss of the ecGCx there is not only disruption of the NVU BBB with paracellular leakage of inflammatory cells but also increased pinocytosis/transcytosis with increased permeability. The PVS/EPVS act as a temporary holding station and checkpoint until the glia limitans is digested or becomes dysfunctional and allows entry into the interstitial spaces of the neuronal parenchyma. *AC(s) = astrocytes; ACef = astrocyte endfeet; ISS = interstitial space(s); MMPs = matrix metalloproteinases; PVS = perivascular spaces; rMGC(s) = reactive microglia cells; rPVMΦ(s) = resident, reactive perivascular macrophages; ROS = reactive oxygen species*.

The ecGCx is a key and critical regulator of permeability, cerebral blood flow, capillary perfusion, and cell adhesion, all of which are necessary for maintenance of CNS homeostasis. Therefore, we should do all we can to maintain the integrity of the ecGCx to decrease the development of EPVS as a result of NVU BBB disruption with increased permeability due to the attenuation and/or loss of the ecGCx. For this reason, the possible mechanisms of how ecGCx perturbations with attenuation and/or loss might result in EPVS are shared (Figure 9). 

The theory that the glia limitans (the basal lamina of perivascular ACef) serve as a second barrier for the passage of proinflammatory leukocytes is substantiated by Horng et al. [70]. 

We now live in one of the oldest global populations ever recorded, that will continue to increase over at least the next two decades until the so-called global ‘baby boomer generation’ has passed [5,20]. Additionally, it is currently known that aging comes with increasing age-related diseases such as cerebrocardiovascular diseases, stroke, impaired cognition, dementias (especially sporadic Alzheimer’s and Parkinson’s disease), and frailty [71]. Furthermore, these age-related diseases are major causes of disability and dependence in elderly individuals that cause a great financial and societal burden [72].

When discussing health with older individuals over the past 30 plus years of the author’s clinical practice in family medicine, three of the most feared diseases are the development of stroke, dementia, and cancer because they so often result in an inability for older people to care of themselves. However, cognitive impairment and dementia are primarily the result of a combination of vascular brain injury and neurodegeneration. Also, vascular injury manifests as a highly prevalent pathologic clinical disease of SVD over a long period time instead of acute onset transient ischemic episodes and strokes [73]. Importantly, these SVD abnormalities are apparent in studies utilizing non-invasive magnetic resonance imaging (MRI) and consist of MRI abnormalities known as white matter hyperintensities, lacunes, microbleeds, microthrombi, and most recently EPVS [74]. Notably, EPVS have been determined to be, and accepted as, biomarkers for SVD [20,31]. In addition to EPVS being biomarkers for SVD, they have also been identified as markers for increased risk of cognitive decline and dementia, independent of other SVD markers [75]. Thus, this is why the author wished to share this emerging accumulation of knowledge regarding the importance of these PVS/EPVS in the growing scientific field of neuroaging. It is estimated that ~ two billion of the global population will be older than 60 years of age by 2050 or the equivalent of 1/5 of the world’s population [76].

Intriguingly, enlarged perivascular spaces, the glymphatic system, and the ecGCx are in their early stages of an exponential growth in the scientific understanding of how they relate to various injurious processes resulting in neuroinflammation. Neuroinflammation and the multiple associated neurologic diseases that affect the brain through neurovascular and neurodegenerative mechanisms have many future questions that need to be asked and answered. It is hoped that this review will provide the stimulus for future research to ask these questions in order to provide the necessary answers. 

## Data Availability

The data and materials can be provided upon reasonable request.

## Abbreviations

AC: astrocyte; ACef, astrocyte endfeet; AGE/RAGE, advanced glycation end products/receptor for advanced glycation end products; AQP4, aquaporin-4; ATIII, antithrombin three; BBB, blood–brain barrier; BEC(s), brain endothelial cell(s); BECact/dys, brain endothelial cell activation/dysfunction; BM, basement membrane; CAV-1, calcium-calmodulin-dependent caveolin-1; CL, capillary lumen; EPVS, enlarged perivascular spaces; GS, glymphatic space; GPI, glycosylphosphatidylinositol HA, hyaluronan; HSPGs, heparan sulfate proteoglycans; ICAM-1, intercellular adhesion molecule-1; ISF, interstitial fluid; ISS, interstitial space; LAN, lanthanum nitrate; La3+, lanthanum plus 3; LOAD, late-onset Alzheimer’s disease; LPS, lipopolysaccharide; MetS, metabolic syndrome; MGCs, microglia cells; MMP-2,-9, matrix metalloproteinase-2,-9; NVU, neurovascular unit; Pc, pericyte; Pcfp, pericyte foot process; PVS, perivascular spaces; PVS/EPVS, perivascular space/enlarged perivascular space; rPVMΦ, resident perivascular macrophages; SAS, subarachnoid space; rPVMΦ, reactive perivascular macrophage; SVD, cerebral small vessel disease; T2DM, type 2 diabetes mellitus; TEM, transmission electron microscopy; TH, thrombomodulin; TIPI, tissue factor pathway inhibitor; TI/AJs, tight and adherens junctions; VAD, vascular artery disease; VCAM-1, vascular cell adhesion molecule-1; WMH, white matter hyperintensities.
